# Defensin–lipid interactions in membrane targeting: mechanisms of action and opportunities for the development of antimicrobial and anticancer therapeutics

**DOI:** 10.1042/BST20200884

**Published:** 2022-01-11

**Authors:** Matthew J. A. Hein, Marc Kvansakul, Fung T. Lay, Thanh Kha Phan, Mark D. Hulett

**Affiliations:** Department of Biochemistry and Genetics, La Trobe Institute for Molecular Science, La Trobe University, Melbourne 3086, Australia

**Keywords:** cancer, defensin, host defence peptide, infection, innate immunity, lipid

## Abstract

Defensins are a class of host defence peptides (HDPs) that often harbour antimicrobial and anticancer activities, making them attractive candidates as novel therapeutics. In comparison with current antimicrobial and cancer treatments, defensins uniquely target specific membrane lipids via mechanisms distinct from other HDPs. Therefore, defensins could be potentially developed as therapeutics with increased selectivity and reduced susceptibility to the resistance mechanisms of tumour cells and infectious pathogens. In this review, we highlight recent advances in defensin research with a particular focus on membrane lipid-targeting in cancer and infection settings. In doing so, we discuss strategies to harness lipid-binding defensins for anticancer and anti-infective therapies.

## Introduction

Host defence peptides (HDPs; also referred to as cationic antimicrobial peptides) are key components of the innate immune system across all kingdoms of life [1,2]. Defensins, a prominent HDP class, are typically cationic, β-sheet and cysteine-rich and maintain conserved disulfide-stabilised structures [3,4]. The arrangement of two specific disulfide bonds in defensins define their classification into either the *cis-* or *trans*-defensin superfamilies, which are evolutionally convergent (see [4,5] for more details). For *cis*-defensins (dominated by plant defensins), the two disulfide bonds are parallel and tether the final β-strand to an α-helix. Conversely, in *trans*-defensins (including animal and human defensins), the two analogous disulfide bonds are orientated in opposite directions from the final β-strand to different secondary structure elements [4,5]. The disulfide bond framework and the functionally important β2–β3 loop between two antiparallel β-strands are highly conserved amongst defensin family members [4,6].

**Figure 1.**
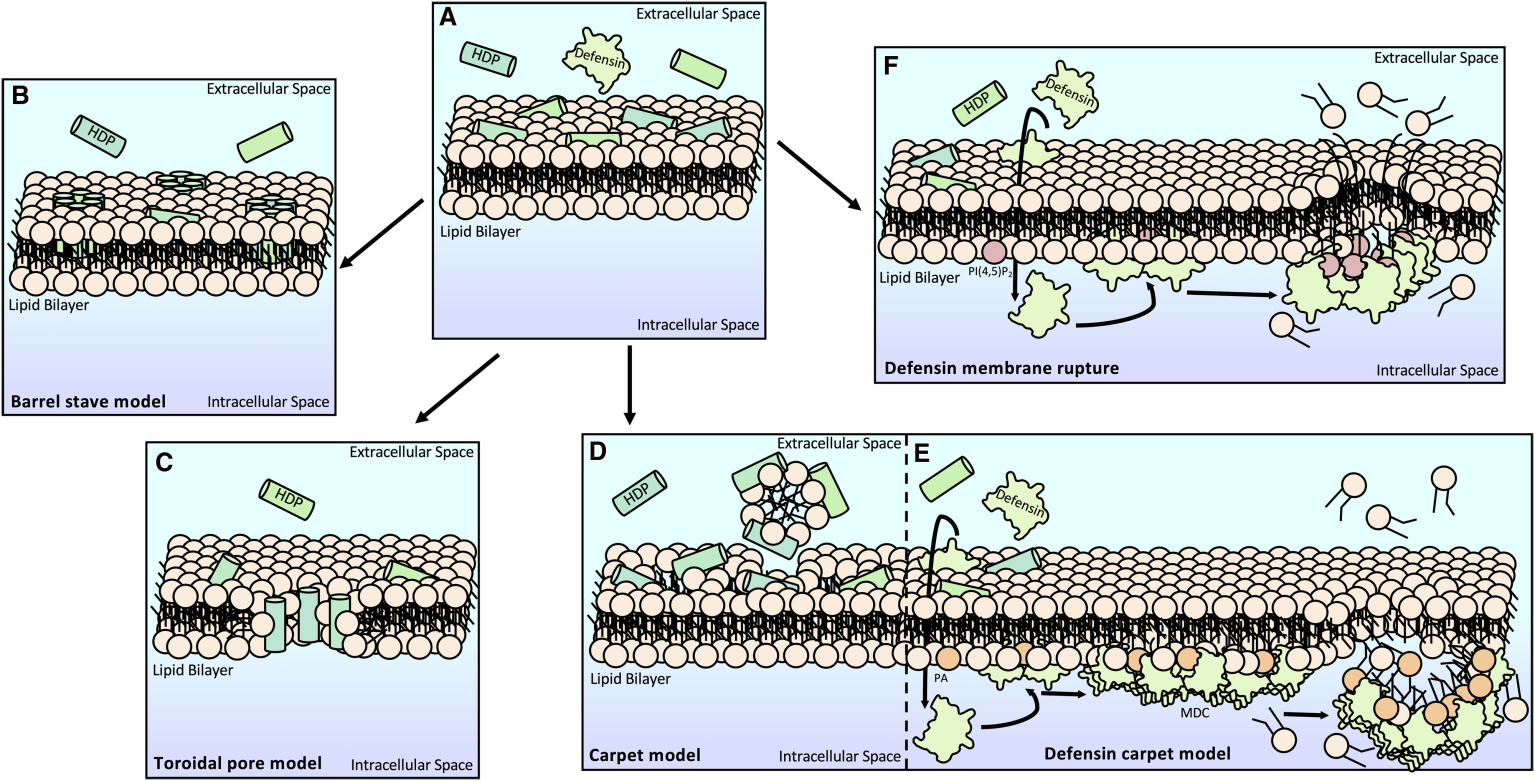
Models of membrane permeabilisation induced by HDPs and defensins. (**A**) HDPs and defensins first associate with the cell membrane via electrostatic charge interactions. (**B**) In the barrel stave model, the HDPs embed themselves through the membrane, forming a pore completely lined with peptide. (**C**) In the toroidal pore model, the HDPs force the lipid membrane to curve and form a continuous lipid layer. The pore is lined with lipid head groups and peptide. (**D**) In the carpet model, the HDPs spread over the surface of the membrane like a surfactant until a critical concentration is reached at which point, micelles are formed that as the membrane breaks apart. (**E**) In the NaD1–PA carpet model, NaD1 monomers cross the membrane before engaging PA and forming dimers that assemble into the MDC. The MDC induces membrane curvature stress and subsequent membrane rupture. (**F**) In the defensin membrane disruption model (as exemplified by NaD1 and NsD7), defensin monomers cross the membrane before engaging PI(4,5)P_2_ and forming dimers that oligomerise into arch-shaped assemblies to induce membrane rupture. The defensins may also act from the extracellular membrane surface in some cases as abnormal cells such as in cancer often demonstrate disrupted membrane asymmetry.

Like other HDPs, many defensins exhibit potent antimicrobial and anticancer activity, with additional roles including but not limited to ion channel blocking and immune modulation [7–9]. These antimicrobial and anticancer effects have largely been attributed to their membrane-permeabilising property (Figure 1A), for which three mechanistic models have been proposed: the barrel stave (Figure 1B), toroidal pore (Figure 1C) and carpet models (Figure 1D) [10–12]. The ornamental tobacco (*Nicotiana alata*) defensin NaD1 in complex with phosphatidic acid (PA) was the first direct structural evidence for the carpet model (Figure 1E) [13]. Intriguingly, emerging evidence suggests a novel membrane targeting and membrane disrupting mechanism, in which defensins including NaD1, human β-defensin 2 (HBD-2) and human β-defensin 3 (HBD-3) can preferentially bind specific phosphoinositides, ultimately leading to membrane permeabilisation in tumour, fungal and bacterial cells (Figure 1F) (elaborated below) [6,14–16].

The potent antimicrobial and anticancer activities of defensins make them attractive candidates for development as novel therapeutics. Indeed, the specific lipid-targeting and membrane-permeabilising activities of defensins have the potential to address some of the key concerns for current antimicrobial and anticancer therapeutics, such as drug/antibiotic resistance and severe off-target effects [2]. In this review, we discuss the current understanding of the molecular interactions of defensins with cell membranes as well as highlight evidence supporting that this process differs from those previously proposed for other HDPs. We also outline the potential utility of these lipid-binding peptides as novel antimicrobial and anticancer agents.

## Lipid binding-mediated membrane permeabilisation by defensins

Recent studies have shown that defensins interact with microbial pathogens and/or tumour cell membranes by binding specific phospholipids to cause membrane permeabilisation [6,15,17–21]. Biochemical, structural and functional evidence for various defensin–lipid interactions have been reported (Table 1), highlighting the conservation of key lipid binding regions in defensins and the overall mechanism that ultimately result in membrane permeabilisation. The first defensin–lipid interactions were demonstrated for bacterial membrane components such as 1-palmitoyl-2-oleoyl-sn-glycero-3-phosphatidylglycerol (POPG), date back to 1994 [14]. However, it was not until recently that the structure–function relationship and detailed mechanisms of these lipid interactions were reported for several plant and human defensins.

**Table 1.** Examples of lipid-binding defensins

HDP	Organism	Lipid	References
Copsin	*Coprinopsis cinerea*	Lipid II	[33]
DmAMP1	*Dahlia merckii*	Sphingolipids	[34]
Eurocin	*Eurotium amstelodami*	Lipid II	[35]
Gallicin	*Mytilus galloprovincialai*	Lipid II	[36]
HBD-2	*Homo sapiens*	PS, PI(3)P; PI(4)P, PI(5)P, PI(3,4)P_2_, PI(3,5)P_2_, PI(4,5)P_2_, PI(3,4,5)P_3_, Cardiolipin, Sulfatide	[18]
HBD-3	*Homo sapiens*	PA, PS, PE, PI(3)P, PI(4)P, PI(5)P, PI(3,4)P_2_, PI(3,5)P_2_, PI(4,5)P_2_, PI(3,4,5)P_3_, Cardiolipin, Sulfatide	[27]
HNP-1	*Homo sapiens*	Palmitoyloleoylphosphatidylglycerol (POPG), Lipid II	[37]
HNP-2	*Homo sapiens*	POPG	[14,38]
HsAPF1	*Heuchera sanguinea*	PA, PI(3,4,5)P_3_, PI(3,4)P_2_	[39]
Lc-def	*Lens culinaris*	POPG	[40]
Lucifensin	*Lucilia sericata*	Lipid II	[36]
MsDef1	*Medcago sativa*	PA, PI(3)P, PI(4)P, PI(5)P, PI(3,4)P_2_, PI(3,5)P_2_, PI(4,5)P_2_, PI(3,4,5)P_3,_ Glucosylceramide	[16]
MtDef4	*Medicago truncatula*	PA	[16]
MtDef5	*Medicago truncatula*	PA; PS; PI(3)P; PI(4)P; PI(5)P;PI(3,5)P_2_; PI(4,5)P_2_	[21]
NaD1	*Nicotiana alata*	PA, PS; PI(3)P, PI(4)P, PI(5)P, PI(3,4)P_2_, PI(3,5)P_2_, PI(4,5)P_2_, PI(3,4,5)P_3_, Cardiolipin, Sulfatide	[15]
NoD173	*Nicotiana occidentalis*	PI(4,5)P_2_	[20]
NsD7	*Nicotiana suaveolens*	PA	[19]
Oryzeacin	*Aspergillus oryzea*	Lipid II	[36]
OsAFP1	*Oryza sativa*	PI(3)P, PI(4)P, PI(5)P, PI(3,5)P_2_, PI(4,5)P_2_	[41]
Plectasin	*Pseudoplectania nigrella*	Lipid II	[36]
Psd1	*Pisum sativum*	Ergosterol, Glycosphingolipid	[42]
Psd2	*Pisum sativum*	Ergosterol, Glucosceramides, Phosphatidylcholine, PI(3)P, PI(5)P, PS	[43]
RsAFP2	*Raphanus sativus*	Glucosylceramides, Sphingolipids	[30]
Sd5	*Saccharum officinarum*	Glucosylceramides	[44]
TPP3	*Solanum lycopersicum*	PI(4,5)P_2_	[17]

**Figure 2.**
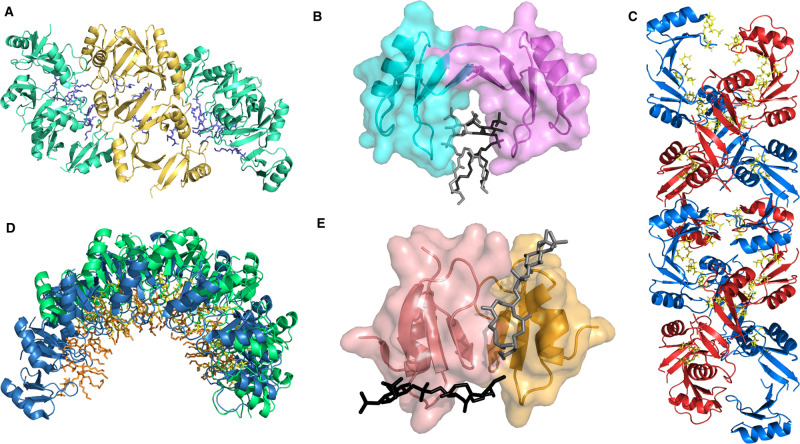
X-ray crystal structures of defensin–lipid complexes. (**A**) The membrane disruption complex formed in a carpet-like model of membrane disruption by NaD1 (PDB: 6B55) engaging PA (three dimer pairs shown in aqua, four dimer pairs shown in gold). (**B**) The conserved cationic grip of an NaD1 dimer (PDB: 4CQK) (monomer 1 in cyan, monomer 2 in pink) binding two PI(4,5)P_2_ molecules (grey and black). (**C**) The oligomer of NsD7–PA (PDB: 5KK4) showing the NsD7 double helix (helix 1 in blue, helix 2 in red). PA (yellow) is bound in between the dimers and in the cationic grip. (**D**) Comparison of NsD7 (PDB: 5VYP) (green) and NaD1 (PDB: 4CQK) (blue) engaging PI(4,5)P_2_ (orange for NaD1, yellow for NsD7), both of which form multimeric arch-shaped oligomers that are proposed to exert torsional strain on the membrane. (**E**) The asymmetric dimer of HBD-2 (PDB: 6CS9) (monomer 1 in salmon, monomer 2 in orange) showing the two PI(4,5)P_2_ binding sites (lipids shown in grey and black). Images generated using PyMOL.

*Medicago truncatula* defensin 4 (MtDef4) has been shown to bind PA and interact with cells via its positively charged β2–β3 loop region. Notably, the substitution of cationic loop residues to alanine residues perturbed both the lipid binding and antifungal activities of MtDef4 [16]. NaD1 also binds to PA as a part of its fungal killing mechanism. X-ray crystallographic analysis showed that the β2–β3 loop was important for lipid binding, and mutagenesis studies confirmed this finding with mutants of proposed key lipid-binding residues showing reduced efficacy against fungal cells [13]. Lipid binding also plays a role in defensin oligomerisation and complex formation, which has been proposed as a key event in the membrane permeabilising activity of some defensins [14,16,20,23]. Intriguingly, the NaD1–PA crystal structure reveals an oligomeric structure, termed the membrane disruption complex (MDC). The MDC is formed via the assembly of groups of defensin dimers (containing either three or four dimer pairs; Figure 2A) with each dimer in a conserved cationic grip configuration engaged with the head group of a single PA molecule (Figure 2B) [13]. The formation of the MDC at the membrane is postulated to generate curvature stress on the membrane, which aids in membrane destabilisation and rupture [13]. Furthermore, this NaD1 MDC appears to engage PA from one side, resembling the carpet model of membrane binding and disruption that has been previously proposed for HDPs (Figure 1E) [13]. Additionally, *Nicotiana suaveolens* defensin 7 (NsD7) adopts a double-helical oligomer upon interaction with PA that was demonstrated by X-ray crystallography (Figure 2C) [19]. Site-directed mutagenesis of key lipid-binding residues, which impaired NsD7 oligomerisation, had a subsequent effect on membrane permeabilisation. This underscores the importance of lipid binding for oligomerisation that is required for defensin membrane targeting [15,19,22].

In addition to PA, NaD1 binds phosphorylated phosphatidylinositols, particularly phosphatidylinositol 4,5 bisphosphate (PI(4,5)P_2_) [15]. Like PA, upon PI(4,5)P_2_ binding, NaD1 dimerises and adopts the same lipid-binding cationic grip dimer (Figure 2B), and the NaD1–PI(4,5)P_2_ crystal structure adopts a distinct arch-shaped higher-order complex. This is also observed during NsD7–PI(4,5)P_2_ binding (Figure 2D) [13,15,22]. These structural studies of NaD1 and NsD7 indicate that defensins can form structurally different oligomeric complexes driven by different phospholipids [22]. Furthermore, these studies indicate that PI(4,5)P_2_ binding-induced tumour cell membrane permeabilisation by NaD1 and NsD7 may be executed through a novel mechanism that differs from either carpet-like or other proposed models. While it is tempting to speculate that defensin arch-shaped complexes may come together to form ‘carpet-like’ configurations on a biological membrane, which of these models is more physiologically relevant remains to be experimentally determined. In any case, the mechanism first necessitates the entry of NaD1 into the tumour cell and binding to PI(4,5)P_2_ at the inner membrane leaflet prior to inducing membrane blebbing and ultimately resulting in cell lysis (Figure 1F) [15]. It should be noted that membranes feature a complex mix of lipids. As such, defensins are likely to interact with multiple lipid targets during membrane rupture.

Defensin uptake into some fungal cells is energy-dependant. When *Candida albicans* was subjected to cold or latrunculin A treatment (to inhibit ATP production and endocytosis, respectively, inhibiting NaD1 internalisation), NaD1-induced cell death was significantly reduced [23]. Additionally, MtDef4 uptake into *Neurospora crassa* and *Fusarium graminearum* was significantly reduced following cold or sodium azide treatment both of which block ATP synthesis required for energy dependant internalisation [24]. These studies suggest that defensins require both active uptake into the cytoplasm and intracellular targeting for their membrane rupturing effect [23,24]. It is worth noting that in some cases such as in tumour cells where membrane asymmetry is often disrupted, defensins may also be able to act on the outer leaflet of the membrane in addition to the requirement for internalisation [25]. Morphologically, tumour cells undergoing NaD1-induced cell death show large necrotic-like membrane blebs, and become permeable to the nucleic acid dye propidium iodide, indicating damage to membrane integrity in a process distinct from apoptosis [26].

The importance of PI(4,5)P_2_ interaction for membrane targeting has also been demonstrated for tomato pistal predominant defensin 3 (TPP3) and HBD-3, which bind exclusively or preferentially to PI(4,5)P_2_ [17,27]. Effectively, TPP3 and HBD-3 deploy a similar mechanism to NaD1 that requires internalisation before binding to intracellular PI(4,5)P_2_ to induce membrane blebbing and tumour cell permeabilisation [17,27]. In further support of the importance of PI(4,5)P_2_-targeting by TPP3 in membrane permeabilisation, the sequestration of PI(4,5)P_2_ by neomycin has been shown to cause dose-dependent inhibition of TPP3-induced cell death. In contrast, neomycin had no effect on the membrane-lysing ability of LL-37, a toroidal pore-forming peptide [18]. These data not only suggest the conserved critical role of PI(4,5)P_2_ in mediating defensin-induced membrane permeabilisation, but also further emphasise a distinct mechanism of specific lipid-targeting by defensins compared with the aforementioned general membrane-binding models of most HDPs. This notion is further supported in a more recent report on HBD-2 that describes how PI(4,5)P_2_ binding is indispensable for its potent antifungal activity [18]. Interestingly, unlike the plant defensins, the HBD-2–PI(4,5)P_2_ crystal structure reveals a uniquely asymmetric conformation with two different lipid-binding sites (Figure 2E), one which is positively charged whilst the other is more hydrophobic and engages the acyl chain of the lipid molecule [18]. Mutations of key lipid-binding residues in either site substantially impede fungal cell killing by HBD-2 [18]. It remains unknown whether the conformational disparity in PI(4,5)P_2_ binding between human and plant defensins is due to the fundamental differences in their tertiary structures. Namely, the orientation of disulfide bonds around a central α-helix that determines their classification into either the *cis* (two disulfide bonds to the α-helix; plant defensins) or *trans* (one disulfide bond to the α-helix; human defensins) defensin superfamily [4,18].

In addition to PI(4,5)P_2_, the binding of other membrane phospholipids by defensins has been shown to mediate the antifungal activity. *M. truncatula* defensin MtDef5, a novel bi-domain defensin, reportedly binds strongly to monophosphorylated phosphoinositol such as phosphatidylinositol 3-phosphate (PI(3)P) and phosphatidylinositol 4-phosphate (PI(4)P) as a part of its mechanism of action against plant bacterial pathogens [6,21]. It is, therefore, implied that MtDef5 has likely evolved multifaceted anti-infective mechanisms involving both membrane targeting and interaction with intracellular targets [6]. Of great interest will be the determination of MtDef5–lipid structures as this will provide valuable insights into how the bi-domains associate and whether they form the lipid-binding cationic grip that is observed for other plant defensins [22].

The bacterial cell wall precursor lipid II is another target of defensins. Lipid II is utilised in the final step of peptidoglycan synthesis and is a target of current antibiotic treatments such as vancomycin [28]. Oyster defensins Cg-Defh1, Cg-Defh2 and Cg-Defm all bind essentially irreversibly to lipid II [28]. Interestingly, the binding of oyster defensins to lipid II-containing liposomes varied among the three defensins tested, and the strength of binding as measured via surface plasmon resonance correlated with the ability to inhibit the growth of *Staphylococcus aureus* [28]. As defensins Cg-Defh1, Cg-Defh2 and Cg-Defm were able to inhibit the growth of Gram-positive but not Gram-negative bacteria, Cg-Defh1, Cg-Defh2 and Cg-Defm are thought to interact with lipid II at the extracellular interface [28].

Glucosylceramide (GluCer), a membrane sphingolipid regulating fungal growth, hyphal formation and fungal virulence, is a key binding partner for many antifungal defensins including RsAFP2 (from *Raphanus sativus*) and *Medicago sativa* defensin 1 (MsDef1) [29–31]. RsAFP2 is selective for fungal GluCer and is unable to bind to the structurally related human GluCer. Fungal strains which lack GluCer or its synthesising enzyme glucosylceramide synthase are resistant to RsAFP2 treatment [29,32]. Unlike many of the defensins listed above, the GluCer-binding RsAFP2 does not appear to form pores to permeabilise membranes, but instead activates downstream pathways that ultimately lead to fungal cell death (details below) [32]. Similarly, GluCer binding also contributes to MsDef1-induced antifungal activity against *F. graminearum*, which also become resistant upon GluCer deficiency [30].

## Mechanisms downstream of lipid binding: more than just membrane disruption

**Figure 3.**
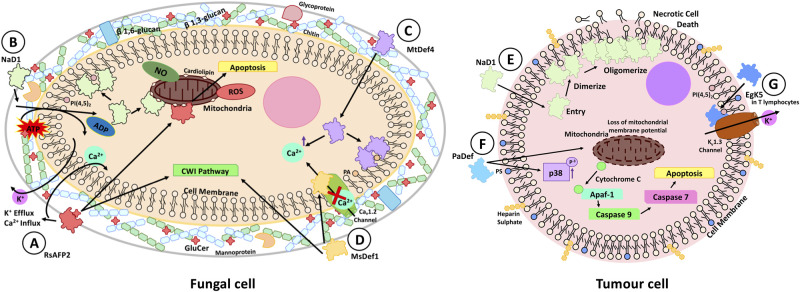
Mechanisms of defensin action downstream of lipid binding. (**A**) Binding of RsAFP2 to GluCer in the membrane of fungi induces the influx of Ca^2+^ and efflux of K^+^ along with activation of the CWI pathway and ROS formation, leading to apoptosis. (**B**) NaD1 kills fungi by a three-step mechanism involving cell wall interaction, energy-dependent import followed by ROS and NO production along with lipid binding and membrane permeabilisation. (**C**) MtDef4 induces dysregulation of Ca^2+^ levels by mechanisms involving GluCer, resulting in dysregulated Ca^2+^ levels. (**D**) MsDef1 blocks Ca^2+^ by interaction with Ca_v_1.2 channels in the membrane. Additionally, MsDef1 is able to induce the activation of the CWI repair pathway. (**E**) NaD1 induces membrane damage and necrotic cell death in tumour cell settings, via first dimerisation and lipid engagement before oligomerisation and membrane rupture. (**F**) PaDef induces the loss of mitochondrial membrane potential and induces apoptosis via caspases 7/9 activation downstream of Apaf-1. PaDef additionally up-regulates the levels of phosphorylated p38. (**G**) Defensin analogue EgK5 is able to bind to PI(4,5)P_2_ in the membrane and cause the rundown of K_v_1.3 channels.

In addition to membrane permeabilisation, other downstream effects of membrane binding by defensins have been suggested, further highlighting their multifaceted mechanisms in combating microbial pathogens and tumour cells. Generally, defensins can trigger different cellular effects including, but not limited to, reactive oxygen species (ROS) and/or nitric oxide (NO) production, activation of cell wall integrity (CWI) pathway and dysregulation of ionic homeostasis, ultimately contributing to cell death (Figure 3) [45].

Defensins NaD1 and RsAPF2 have both been shown to induce the formation of ROS (and NO in the case of NaD1) in fungal cells, hence significantly damaging key cellular components and processes [45,46] (Figure 3A,B). RsAFP2 is believed to cause induction of ROS as a downstream signal from its binding to GluCer in the membrane. This increase in intracellular ROS is believed to induce apoptosis in yeast which is also observed upon RsAFP2 treatment (Figure 3A) [47]. NaD1 induces ROS and NO as the final step in a three-step mechanism of action against fungal cells. Initiation of the process involves interactions with cell wall components such as glycosylated proteins or 1,3-β-glucan, which drives energy-dependant import (step 2), allowing lipid binding and ROS/NO production (step 3) (Figure 3B) [15,23,48,49]. The induction of ROS/NO by NaD1 occurs via interaction with yeast mitochondria, as *Saccharomyces cerevisiae* with an inactive mitochondria respiratory chain are more resistant to NaD1 than wild-type fungi [48]. NaD1 binds cardiolipin (an abundant mitochondrial inner membrane lipid) which, along with the mitochondrial respiratory chain components in yeast, may provide explanation of the mechanism of ROS/NO generation by NaD1 [15,48,50].

In addition to its ability to induce cellular ROS and apoptosis, RsAFP2 interaction with GluCer in the membrane is able to induce the efflux of K^+^ and influx of Ca^2+^, thus disturbing the homeostasis of cellular ion concentrations (Figure 3A) [51]. Additionally, both *Medicago* defensins MsDef1 and MtDef4 reportedly induce the dysregulation of homeostatic Ca^2+^ level (Figure 3C,D) [52,53]. A study comparing Ca^2+^ modulation by both MsDef1 and MtDef4 in *N. crassa* reported a significant decrease in Ca^2+^ amplitude compared with mechanical perturbation, with defensin-treated fungi also failing to restore resting Ca^2+^ levels [53]. Interestingly, MtDef4 treatment of a *N. crassa* Δ*gcs* mutant (lacking GluCer synthase) no longer reduced Ca^2+^ amplitude when compared with mechanical perturbation indicating a role for GluCer in MtDef4 Ca^2+^ modulation (Figure 3C) [53]. MsDef1 can block the L-type calcium channel Ca_v_1.2 with MsDef1 treatment blocking up to 90% of Ca^2+^ current (Figure 3D). An Arg-38 residue at the base of the β2–β3 loop was shown to be important for the Ca_v_1.2 channel blocking ability by MsDef1 [52]. The β2–β3 loop is also important for defensin lipid binding, as MsDef1 in addition to GluCer binding can bind phospholipids such as PA and PI(4,5)P_2_ [16]. Studies investigating cofactors of ion channels reveal an important role for PI(4,5)P_2_ in channel stabilisation and activation [9]. Whilst not shown experimentally it is tempting to speculate MsDef1 may block the Ca_v_1.2 channel via a lipid-dependant mechanism, which has been shown for the defensin EgK5 (discussed later) [9].

Various cell signalling pathways have been shown to be activated in response to defensin exposure. MsDef1 and RsAFP2 both induce increased MAPK signalling and activation of the CWI pathway in response to damage caused by defensins binding to GluCer in the membrane (Figure 3A,D) [45]. RsAFP2 induces increased phosphorylation of Mkc1p, a downstream interaction partner of Pkc1p both involved in the CWI pathway in *C. albicans* (Figure 3A) [32]. *F. graminearum* mutants lacking *MGV*1 (a gene involved in the CWI pathway in *F. graminearum*) are significantly more sensitive to MsDef1 than wild type *F. graminearum.* The increased sensitivity is believed to be caused due to decreased signalling through the Mgv1 MAPK signalling cascade (Figure 3D) [30].

The downstream mechanisms of defensins against tumour cells include activation of classical cell death pathways such as apoptosis and necrosis (Figure 3E–G) [26,54]. NaD1 induces necrotic cell death as a result of membrane rupture (Figure 3G) [26]. It is likely that under subacute NaD1 treatment (<10 µM) tumour cells would induce activation of membrane repair mechanisms such as micro-particle shedding (in an effort to shed the defensin damaged areas), patch-mediated repair and blebbing [55] (see [55] for a comprehensive review of plasma membrane repair mechanisms). However, prolonged exposure to NaD1 is likely to overwhelm such mechanisms and thus render the cell non-viable. Interestingly, in contrast to NaD1, breast cancer cells (MCF-7) treated with PaDef (from avocado fruit) showed activation of the intrinsic apoptotic pathway with up-regulation of caspase 7/9 genes, along with cytochrome c and Apaf-1 (Figure 3F) [54]. Additionally, PaDef also induces loss of mitochondrial membrane potential and increases the phosphorylation of p38 (Figure 3F) [54], which is involved in proliferation and differentiation along with cell stress responses, especially metabolic, oxidative and endoplasmic reticulum stress responses [56]. A designed defensin analogue EgK5, which similarly to natural defensins such as NaD1, is able to bind lipids in the plasma membrane, also binds to the potassium channel K_v_1.3 in transformed T lymphocytes [9]. Binding of K_v_1.3 in the membrane by EgK5 induces a current rundown of the channel, via a PI(4,5)P_2_ dependent mechanism in which EgK5 depletion of PI(4,5)P_2_ (a cofactor for K_v_1.3) triggers the K_v_1.3 channel to release K^+^ (Figure 3G) [9].

## Developing lipid-targeting defensins as novel anticancer and anti-infective therapeutics

Infectious diseases and cancer remain urgent public health and medical issues. The continued emergence of new infectious agents and multidrug/antibiotic resistance is of particular concern [57,58]. Many drugs currently under development exhibit similar mode(s) of action to traditional drugs, which could make them vulnerable to the same resistance mechanisms [59,60]. Therefore, the specific lipid-targeting and potent membrane-permeabilising properties of defensins provide an exciting avenue for anticancer and anti-infective therapeutic design. Advantages of such treatments could include reduced susceptibility to resistance due to the targeting of very conserved cellular features, increased specificity for infectious pathogens and tumour cells, and the ability to target metabolically active and dormant tumour cells [2,61–63]. Furthermore, some defensins are potent at low micromolar concentration ranges against a broad spectrum of tumour cells and pathogens *in vitro* and *in vivo*, including multidrug-resistant bacteria [2,8,16]. Additionally, due to their small compact size and high disulfide content, defensins are stable to protease degradation [15,23,48].

As detailed above, defensins bind a wide range of lipids from both prokaryotic and eukaryotic organisms, speaking to the diversity of structures and functions within this family. Not surprisingly, defensins from the same species can have different lipid binding profiles and downstream mechanisms of action, likely to have arisen as a result of selective pressure to protect hosts from various pathogens [64]. In the context of human health and disease, the role of lipids is extensive but is often poorly understood. For diseases including cancer, Alzheimer's disease and liver disease, lipids represent important biomolecules for disease progression and resolution [65–67]. Additionally, various phosphoinositides are implicated in the establishment and progression of pathogenic infections [65]. Microbial pathogens are able to modulate the regulation of various phosphoinositides (including PI(4,5)P_2_, phosphatidylinositol 3,4,5-trisphosphate (PI(3,4,5)P_3_), PI(3)P and PI(4)P) in order to modulate host cell functions. These include roles in phagocytosis, membrane ruffling and cup formation, phagosomal lysis and fusion, and the modulation of endoplasmic reticulum machinery, respectively [65]. Thus, defensin-based therapeutics may offer opportunities to directly target pathogens as well as control disease progression through their lipid mediators.

In the next section, defensins and their potential applications for targeting microbial pathogenesis and cancer through lipid interactions are discussed along with limitations currently restricting defensins for therapeutic applications.

## Defensins as therapeutics against antibiotic-resistant microbes

Antibiotics revolutionised disease treatment by targeting key bacterial cell processes and allowing selectivity from host tissue [62], however, their misuse has led to the rapid emergence of multidrug-resistant microbes [8]. As discussed above, defensins are unique in that they are able to specifically target specific membrane phospholipids, enabling them to kill microorganisms that are otherwise resistant to other forms of antimicrobials [68]. Furthermore, defensin-based therapeutics are less susceptible to resistance mechanisms than traditional therapeutics. In a study testing the development of *S. cerevisiae* resistance to NaD1 treatment, the authors showed that resistance was developed more slowly compared with the antifungal compound, caspofungin [63]. Furthermore, multiple genome regions in *S. cerevisiae* were identified to contribute to resistance, with resistant strains requiring multiple mutations for resistance. However, the formation of resistance also resulted in decreased cellular fitness as indicated by growth speed and size when compared with wild-type cells [63].

*In vitro* antimicrobial activity has been shown for several defensins including mouse α-defensins Crp-4, rhesus monkey defensin RMAD-4 and θ-defensin RTD-1 from macaques [56]. As little as 3 μM of defensins Crp-4, RMAD-4 or RTD-1 was sufficient for potent antibacterial activity against methicillin-resistant *S. aureus* (MRSA), vancomycin-resistant *S. aureus* strains and ciprofloxacin-resistant *Pseudomonas aeruginosa* [56]*.* Mechanistically, hydrophobic residues are important for the antibacterial activity of Crp-4 and RMAD-4 as mutagenesis of key hydrophobic residues resulted in decreased antibacterial activity compared with wild-type controls [68,69].

The defensin rRpdef1α from manila clam shows activity against both *Escherichia coli* and its biofilms, and is believed to act via mechanisms involving both targeting of extracellular ligands (such as lipopolysaccharide (LPS) and glucan) as well as membrane disruption caused by pore formation [70]. Furthermore, PaDef is active against human bacterial pathogens *E. coli* and *S. aureus* [71]. In addition to antibacterial activity, many plant and human defensins have been characterised to have activity against human fungal pathogens such as *C. albicans*, including NaD1 and HBD-2 via the mechanisms discussed above [18,48]*.* The common bean defensin PvD1 is active against pathogenic fungi *C. albicans*, *Candida buinensis*, *Candida tropicalis* and *Candida parapsilosis* at low micromolar concentrations [72]. PvD1 was also tested *in vivo* in a *C. albicans* infection model of *Gallieria mellonella* (greater wax moth), revealing that treatment with PvD1 significantly increased survival of *G. mellonella* upon infection with various *Candida* strains [72].

Defensins including rRpDef1α, HBD-3, PsD1, HsAFP1 and HsAPF2 are active against bacterial and fungal biofilms [70,73–75]. This defensin-mediated anti-biofilm activity may have applications in the field of medical device implants and prosthetics where infection caused by the formation of biofilms on prosthetic surfaces is of increasing concern [76,77]. HBD-3 reduces the adhesion and formation of MRSA as well as *Staphylococcus epidermidis* and methicillin-resistant *S. epidermidis* (MRSE) biofilms on a titanium surface. Additionally, HBD-3 showed the potential to clear pre-existing MRSA and MRSE biofilms from orthopaedic implants [75].

A few defensins are currently undergoing development and trial for clinical use as treatments for various fungal, bacterial, and viral infections (Table 2). A derivative of a plant defensin, Pezadeftide (previously HXP124) developed by Hexima Limited (Melbourne, Australia) is showing promise as a topical treatment for fungal nail disease following phase IIa clinical trials (ACTRN12618000131257). Brilacidin (formally PMX-30063), a synthetic defensin derivative, is currently undergoing an FDA-fast tracked clinical trials (NCT02324335; NCT01211470) for the treatment of oral mycosis (in patients with head and neck cancer). Brilacidin is also being investigated as a topical treatment for ulcerative proctitis, ulcerative proctosigmoiditis, and an intravenous treatment for acute bacterial skin infections (NCT02052388). Additionally, following successful preclinical trial demonstrating its ability to inhibit SARS-CoV-2 in cell culture [78], Brilacidin has been FDA fast tracked to phase II clinical trials as an intervention for hospitalised COVID-19 patients (NCT04784897). Currently, although there are a limited number of defensins undergoing clinical trial, a number are in preclinical stages of development. Some challenges currently restrict the application of defensins as therapeutics including a limited therapeutic window, high production costs and issues with delivery and formulation [77]. Additionally, further considerations such as peptide stability, bioavailability and target specificity in biological systems are all challenges to be overcome to aid the progression of defensins into clinical trials [77]. However, more defensin-based therapeutics are likely to soon enter clinical trials on the basis of promising pre-clinical efficacy. Once such example is a defensin variant Plectasin, also known as NZ2114. Plectasin has undergone preclinical trials for the treatment of *Streptococcus pneumoniae* and *S. aureus* in a murine infection model, and has shown significant promise as a therapeutic [79]. Optimistically, defensin-based therapeutics, such as Plectasin, will join a host of other HDPs currently under clinical trial (extensively reviewed by Mookherjee et al. [77]) fulfilling their therapeutic potential and gaining real-life application for a range of clinical pathologies.

**Table 2.** Defensins under development for clinical applications

Compound	Defensin	Trial Number	Application	Phase	Company	Outcomes
Pezadeftide (formerly HXP124)	Plant defensin	ACTRN12618000131257	Fungal nail disease	Phase IIa (complete); Stage IIb (ongoing)	Hexima Limited	Excellent clinical efficacy Safe and well tolerated 2-fold higher mycological cure rate than current treatments
Brilacidin (formerly PMX-30063)	Synthetic defensin derivative	NCT02324335; NCT01211470	Oral mucositis in patients with head and neck cancer	Phase 2 complete	Innovation Pharmaceuticals, Inc.	High potential as preventative treatment
		NCT02052388	Acute bacterial skin and skin structure infections	Phase 2 complete, Phase 3 planned	Innovation Pharmaceuticals, Inc.	Single dose equivalent in safety and efficacy to a 7-day antibiotic regimen
		NCT04784897	COVID-19 hospitalised infections	Phase 2 complete	Innovation Pharmaceuticals, Inc.	To determine safety and efficacy for COVID-19 treatment
Plectasin (also known as NZ2114)	Defensin variant	N/A	Treatment of Gram-positive infections	Pre-clinical	Novozymes	Effective against *Streptococcus pneumococcus* Effective in reducing CSF bacterial concentration

## Defensin-based treatment against tumours

Cancer is a disease with high morbidity and mortality and many current treatment options have side effects due to toxicity towards healthy cells [2,80–83]. Additionally, the emergence of secondary treatment-related cancers is of growing concern [80,84]. Tumour cells undergo various changes to their lipid expression profiles that make them susceptible to treatment with defensins [20]. For example, increased levels of PI and its phosphoinositide derivatives are well-reported during tumourigenesis, associated with tumour growth, proliferation and metastasis. Phosphatidylinositol 5-phosphate (PI(5)P) and its metabolising enzyme PIKfyve have been shown to increase the rate of tumour cell migration, via an increase in cellular PI(5)P, which results in increased activation of Rac1 via recruitment of effectors to PI(5)P [85]. Additionally, phosphatidylinositol 3,4 bisphosphate (PI(3,4)P_2_) via localisation to invadopodium enhances tumour cell migration whilst PI(4,5)P_2_ influences the invasiveness, migration, cell polarity and metastasis of tumour cells via its many effectors [65,85]. Furthermore, the dysregulation of PI(3)K via activating mutations increases PI3K-Akt-mTOR pathway flux, thus promoting cancer growth and survival as well as cell polarity driven epithelial-to-mesenchymal transition [65,86]. Tumour cell plasma membrane levels of PA are also elevated due to changes in cell metabolism including increased flux via EGRF receptors and G protein-dependant activation of phospholipase D, which is involved in PA biosynthesis. Increased PA levels, in turn, activate kinases such as MAPK and ABL tyrosine kinase 1 which are implicated in cancer progression [87]. As a result of tumourigenesis-induced dysregulation of lipid-transporting enzymes (e.g. flippases, floppases, scramblases, aminolipid translocase), phosphatidylserine (PS) and phosphatidylethanolamine (PE) are shuffled to the outer leaflet of the tumour cell plasma membrane [87,88]. In cancers such as colorectal and metastatic liver cancer, phospholipid scramblase 1 (causes bidirectional membrane lipid scrambling), is up-regulated and thought to be responsible for a breakdown of membrane asymmetry [89,90]. The dysregulation tumour cell membrane composition has been shown in a tumour implantation model of Hodgkins lymphoma in SCID mice which Annexin V and a monoclonal antibody 9D2 (which specifically recognises anionic lipids) localised to the vascular endothelium in tumours but not normal endothelium, indicating increased exposure of anionic lipids in the membranes of tumour endothelium [91]. This may aid in sensitising the cells to defensin treatment whilst in a dormant or actively dividing state [25,90]. Furthermore, a study of tumourigenesis in *Drosophila* showed that tumour necrosis factor (TNF) caused the exposure of PS in tumour cells which made them selectively permeable to *Drosophila* Defensin. This study reported that the defensin bound to PS-rich regions in the tumours which results in cell death and tumour regression [92]. These data indicate that increased negative charge on tumour cell membranes may cause them to be more susceptible to defensin attack and permeabilisation.

Many defensins have been shown to be active against a wide variety of tumour cell lines *in vitro*. Examples include NaD1 against human colon cancer HCT-116, breast cancer MCF-7, melanoma MM170 and cervical HeLa cancer cells [15]; TPP3 against human monocytic lymphoma U937 [17]; PaDef against MCF-7 cells and myeloid leukaemia K562 cells [54,93]; HBD-3 against U937, HeLa, prostate PC3, leukaemia HL-60 and T-cell leukaemia Jurkat cells [27]; and PvD1 against brain cancer HBMEC and breast cancer MDA-MB-231 cells [94]. However, to date, there is very limited *in vivo* evidence accompanying these *in vitro* studies with the focus of the field tending towards the discovery of new defensins instead of further developing currently known defensins. The plant defensin NoD173 from *Nicotiana occidentalis* (Australian tobacco) has demonstrated *in vivo* activity, dramatically inhibiting the growth of established solid B16-F1 melanoma tumours in a C57BL/6 mouse model. When NoD173 was administered to mice intratumorally at 5 mg/kg body weight (three times per week over 2 weeks), tumour growth was significantly perturbed when compared with both the vehicle control and a chemically inactive form (by reduction and alkylation) of NoD173 [20].

Clinically, there are many opportunities for the use of defensins as anti-infective and cancer therapeutics but much work is still required in this area, including studies on bioavailability, pharmacokinetics, dosing and stability [20]. There are currently some concerns regarding the systemic administration of defensins, which are likely to require yet to be developed delivery systems to target cancer effectively [77]. Nevertheless, nanotechnology-based delivery systems are showing promise in addressing current defensin delivery concerns [95]. In addition to using defensins to directly treat cancer, defensins could be used in conjunction with current chemotherapeutic options to aid in tumour targeting and killing. An example of this approach was reported for the defensin MsDef1 and doxorubicin against triple-negative breast cancer cells (MDA-MB-231R) and oestrogen receptor-positive cells (MCF-7R). In this study, defensin treatment synergistically improved doxorubicin effectiveness [96]. Furthermore, defensins could be used to aid in protection against opportunistic infections by ‘supplementing’ components of the innate immune system during chemotherapeutic treatment [97]. As more research is published on defensin immune-modulatory functions, novel therapeutic opportunities may become apparent for cancer and other immune-related diseases, such as the treatment of inflammatory bowel disease by HBD-2 [98,99].

## Outstanding limitations to be addressed

Despite the promise of using defensins as anticancer and antimicrobial drugs, none are currently approved for clinical use, although several are in clinical trial [77]. One key challenge that has been identified with the use of defensins as treatments for a range of human conditions is their reduced (and in many cases abolished) activity at physiological salt concentration [100,101]. A recent study on a highly charged corn defensin (ZmD32) showed that it was able to retain activity in the presence of salt concentrations as high as 100 mM, compared with other plant defensins such as NaD1 and NaD2 that lose their activity, although the kinetics of ZmD32 killing was reduced in high salt conditions [100].

Defensins often suffer from non-superior efficacy when compared with traditional treatments, potentially due to the inability to deliver defensins therapeutically [77]. Currently, *in vivo* studies typically utilise a subcutaneous injection to deliver defensins [20,98] which is less desirable clinically when compared with oral delivery mechanisms [102]. A novel delivery mechanism is desirable for defensins to address systemic delivery concerns as well as reduce potential toxicities, allowing the defensins to only be exposed to the host cellular environment upon arrival at the target site [103]. Such a delivery system may aid in increasing the bioavailability of defensins, thus making them an incredibly attractive area of research for the defensin field [103].

## Future perspectives and concluding remarks

Defensins represent an armoury of potential anticancer and anti-infective therapeutics, with their unique ability to bind to specific lipids within the target cell membrane, resulting in the permeabilisation of the target membrane and activation of the downstream process which eventuate in cell death. As discussed above, recent studies show the preclinical efficacy of defensins in killing a wide range of human tumour cells, fungal pathogens and antimicrobial-resistant bacteria, along with reduced susceptibility to resistance. However, there are still several challenges to be addressed including delivery mechanisms, potential toxicity and bioavailability. These areas of research provide an opportunity to further advance the field. Future studies investigating both the action of defensins *in vivo*, including pharmacodynamic, bioavailability and efficacy of defensins in treating both microbial and cancer disease models in animals (or other model organisms) would prove beneficial. Together with careful evaluation of the outcomes of current clinical trials, this will provide hope that defensins can one day be used as a new arsenal against pathogens and cancer in clinical settings.

## Perspectives

- The mechanism(s) of membrane interaction by defensins has been of significant interest in the HDP field. Recent research shows that the mechanism of action of defensins may differ to the models traditionally proposed for HDPs.
- Defensins bind membrane lipids via novel mechanisms, which may pave the way to a novel class of antimicrobial and anticancer peptides.
- Defensins present an untapped natural reservoir of novel antimicrobial and cancer therapeutics. Whilst there are currently limitations to their clinical use, research overcoming these limitations may provide a new class of lipid-targeting therapeutics for clinical application.

## Open Access

Open access for this article was enabled by the participation of La Trobe University in an all-inclusive *Read & Publish* pilot with Portland Press and the Biochemical Society under a transformative agreement with CAUL.

## Abbreviations

CWI: cell wall integrity
HBD-2: human beta-defensin 2
MDC: membrane disruption complex
MRSA: methicillin-resistant *S. aureus*
MRSE: methicillin-resistant *S. epidermidis*
PA: phosphatidic acid
PE: phosphatidylethanolamine
PS: phosphatidylserine
ROS: reactive oxygen species
TPP3: tomato pistil predominant defensin 3

## References

[1] Le, C.F., Fang, C.M. and Sekaran, S.D. (2017) Intracellular targeting mechanisms by antimicrobial peptides. Antimicrob. Agents Chemother. 61, e02340-16 10.1128/AAC.02340-16 28167546 PMC5365711

[2] Baxter, A.A., Lay, F.T., Poon, I.K.H., Kvansakul, M. and Hulett, M.D. (2017) Tumor cell membrane-targeting cationic antimicrobial peptides: novel insights into mechanisms of action and therapeutic prospects. Cell Mol. Life Sci. 74, 3809–3825 10.1007/s00018-017-2604-z 28770291 PMC11107634

[3] Lay, F.T., Mills, G.D., Poon, I.K.H., Cowieson, N.P., Kirby, N., Baxter, A.A. et al. (2012) Dimerization of plant defensin NaD1 enhances its antifungal activity. J. Biol. Chem. 287, 19961–19972 10.1074/jbc.M111.331009 22511788 PMC3370180

[4] Shafee, T.M.A., Lay, F.T., Hulett, M.D. and Anderson, M.A. (2016) The defensins consist of two independent, convergent protein superfamilies. Mol. Biol. Evol. 33, 2345–2356 10.1093/molbev/msw106 27297472

[5] Shafee, T.M.A., Lay, F.T., Phan, T.K., Anderson, M.A. and Hulett, M.D. (2017) Convergent evolution of defensin sequence, structure and function. Cell Mol. Life Sci. 74, 663–682 10.1007/s00018-016-2344-5 27557668 PMC11107677

[6] Velivelli, S.L.S., Islam, K.T., Hobson, E. and Shah, D.M. (2018) Modes of action of a Bi-domain plant defensin MtDef5 against a bacterial pathogen *Xanthomonas campestris*. Front. Microbiol. 9, 934 10.3389/fmicb.2018.00934 29867843 PMC5964164

[7] Hancock, R.E.W., Haney, E.F. and Gill, E.E. (2016) The immunology of host defence peptides: beyond antimicrobial activity. Nat. Rev. Immunol. 16, 321–334 10.1038/nri.2016.29 27087664

[8] Lewies, A., Du Plessis, L.H. and Wentzel, J.F. (2019) Antimicrobial peptides: the Achilles’ heel of antibiotic resistance? Probiotics Antimicrob. Proteins 11, 370–381 10.1007/s12602-018-9465-0 30229514

[9] Ong, S.T., Bajaj, S., Tanner, M.R., Chang, S.C., Krishnarjuna, B., Ng, X.R. et al. (2020) Modulation of lymphocyte potassium channel K_V_1.3 by membrane-penetrating, joint-targeting immunomodulatory plant defensin. ACS Pharmacol. Transl. Sci. 3, 720–736 10.1021/acsptsci.0c00035 32832873 PMC7432667

[10] Brogden, K.A. (2005) Antimicrobial peptides: pore formers or metabolic inhibitors in bacteria? Nat. Rev. Microbiol. 3, 238–250 10.1038/nrmicro1098 15703760

[11] Huan, Y., Kong, Q., Mou, H. and Yi, H. (2020) Antimicrobial peptides: classification, design, application and research progress in multiple fields. Front. Microbiol. 11, 2559 10.3389/FMICB.2020.582779/BIBTEX PMC7596191 33178164

[12] Kumar, P., Kizhakkedathu, J.N. and Straus, S.K. (2018) Antimicrobial peptides: diversity, mechanism of action and strategies to improve the activity and biocompatibility in vivo. Biomolecules 8, 4 10.3390/biom8010004 PMC5871973 29351202

[13] Järvå, M., Lay, F.T., Phan, T.K., Humble, C., Poon, I.K.H., Bleackley, M.R. et al. (2018) X-ray structure of a carpet-like antimicrobial defensin-phospholipid membrane disruption complex. Nat. Commun. 9, 1962 10.1038/s41467-018-04434-y 29773800 PMC5958116

[14] Wimley, W.C., Selsted, M.E. and White, S.H. (1994) Interactions between human defensins and lipid bilayers: evidence for formation of multimeric pores. Protein Sci. 3, 1362–1373 10.1002/pro.5560030902 7833799 PMC2142938

[15] Poon, I.K.H., Baxter, A.A., Lay, F.T., Mills, G.D., Adda, C.G., Payne, J.A.E. et al. (2014) Phosphoinositide-mediated oligomerization of a defensin induces cell lysis. eLife 2014, e01808 10.7554/eLife.01808 PMC3968744 24692446

[16] Sagaram, U.S., El-Mounadi, K., Buchko, G.W., Berg, H.R., Kaur, J., Pandurangi, R.S. et al. (2013) Structural and functional studies of a phosphatidic acid-binding antifungal plant defensin MtDef4: identification of an RGFRRR motif governing fungal cell entry. PLoS One 8, 82485 10.1371/journal.pone.0082485 PMC3853197 24324798

[17] Baxter, A.A., Richter, V., Lay, F.T., Poon, I.K.H., Adda, C.G., Veneer, P.K. et al. (2015) The tomato defensin TPP3 binds phosphatidylinositol (4,5)-bisphosphate via a conserved dimeric cationic grip conformation to mediate cell lysis. Mol. Cell. Biol. 35, 1964–1978 10.1128/mcb.00282-15 25802281 PMC4420927

[18] Järvå, M., Phan, T.K., Lay, F.T., Caria, S., Kvansakul, M. and Hulett, M.D. (2018) Human -defensin 2 kills *Candida albicans* through phosphatidylinositol 4,5-bisphosphate-mediated membrane permeabilization. Sci. Adv. 4, eaat0979 10.1126/sciadv.aat0979 30050988 PMC6059731

[19] Kvansakul, M., Lay, F.T., Adda, C.G., Veneer, P.K., Baxter, A.A., Phan, T.K. et al. (2016) Binding of phosphatidic acid by NsD7 mediates the formation of helical defensin-lipid oligomeric assemblies and membrane permeabilization. Proc. Natl Acad. Sci. U.S.A. 113, 11202–11207 10.1073/pnas.1607855113 27647905 PMC5056070

[20] Lay, F.T., Ryan, G.F., Caria, S., Phan, T.K., Veneer, P.K., White, J.A. et al. (2019) Structural and functional characterization of the membrane-permeabilizing activity of *Nicotiana occidentalis* defensin NoD173 and protein engineering to enhance oncolysis. FASEB J. 33, 6470–6482 10.1096/fj.201802540R 30794440

[21] Islam, K.T., Velivelli, S.L.S., Berg, R.H., Oakley, B. and Shah, D.M. (2017) A novel bi-domain plant defensin MtDef5 with potent broad-spectrum antifungal activity binds to multiple phospholipids and forms oligomers. Sci. Rep. 7, 1–13 10.1038/s41598-017-16508-w 29170445 PMC5700942

[22] Järvå, M., Lay, F.T., Hulett, M.D. and Kvansakul, M. (2017) Structure of the defensin NsD7 in complex with PIP_2_ reveals that defensin: lipid oligomer topologies are dependent on lipid type. FEBS Lett. 591, 2482–2490 10.1002/1873-3468.12761 28741756

[23] Hayes, B.M.E., Bleackley, M.R., Anderson, M.A. and van der Weerden, N.L. (2018) The plant defensin NaD1 enters the cytoplasm of *Candida albicans* via endocytosis. J. Fungi. 4, 20 10.3390/jof4010020 PMC5872323 29415460

[24] El-Mounadi, K., Islam, K.T., Hernández-Ortiz, P., Read, N.D. and Shah, D.M. (2016) Antifungal mechanisms of a plant defensin MtDef4 are not conserved between the ascomycete fungi *Neurospora crassa* and *Fusarium graminearum*. Mol. Microbiol. 100, 542–559 10.1111/mmi.13333 26801962

[25] Baxter, A.A., Hulett, M.D. and Poon, I.K. (2015) The phospholipid code: a key component of dying cell recognition, tumor progression and host-microbe interactions. Cell Death Differ. 22, 1893–1905 10.1038/cdd.2015.122 26450453 PMC4816099

[26] Baxter, A.A., Poon, I.K.H. and Hulett, M.D. (2017) The plant defensin NaD1 induces tumor cell death via a non-apoptotic, membranolytic process. Cell Death Discov. 3, 16102 10.1038/cddiscovery.2016.102 28179997 PMC5253418

[27] Phan, T.K., Lay, F.T., Poon, I.K.H., Hinds, M.G., Kvansakul, M. and Hulett, M.D. (2016) Human β-defensin 3 contains an oncolytic motif that binds PI(4,5)P_2_ to mediate tumour cell permeabilisation. Oncotarget 7, 2054–2069 10.18632/oncotarget.6520 26657293 PMC4811302

[28] Schmitt, P., Wilmes, M., Pugnière, M., Aumelas, A., Bachère, E., Sahl, H.G. et al. (2010) Insight into invertebrate defensin mechanism of action: oyster defensins inhibit peptidoglycan biosynthesis by binding to lipid II. J. Biol. Chem. 285, 29208–29216 10.1074/jbc.M110.143388 20605792 PMC2937951

[29] Thevissen, K., Warnecke, D.C., François, I.E.J.A., Leipelt, M., Heinz, E., Ott, C. et al. (2004) Defensins from insects and plants interact with fungal glucosylceramides. J. Biol. Chem. 279, 3900–3905 10.1074/jbc.M311165200 14604982

[30] Ramamoorthy, V., Zhao, X., Snyder, A.K., Xu, J.R. and Shah, D.M. (2007) Two mitogen-activated protein kinase signalling cascades mediate basal resistance to antifungal plant defensins in *Fusarium graminearum*. Cell Microbiol. 9, 1491–1506 10.1111/j.1462-5822.2006.00887.x 17253976

[31] Rochetti, V.P., Rollin-Pinheiro, R., de Oliveira, E.B., da Silva Xisto, M.I.D. and Barreto-Bergter, E. (2020) Glucosylceramide plays a role in fungal germination, lipid raft organization and biofilm adhesion of the pathogenic fungus *Scedosporium aurantiacum*. J. Fungi. 6, 1–16 10.3390/jof6040345 PMC7762401 33302332

[32] Thevissen, K., De Mello Tavares, P., Xu, D., Blankenship, J., Vandenbosch, D., Idkowiak-Baldys, J. et al. (2012) The plant defensin RsAFP2 induces cell wall stress, septin mislocalization and accumulation of ceramides in *Candida albicans*. Mol. Microbiol. 84, 166–180 10.1111/j.1365-2958.2012.08017.x 22384976 PMC3405362

[33] Essig, A., Hofmann, D., Münch, D., Gayathri, S., Künzler, M., Kallio, P.T. et al. (2014) Copsin, a novel peptide-based fungal antibiotic interfering with the peptidoglycan synthesis. J. Biol. Chem. 289, 34953–34964 10.1074/jbc.M114.599878 25342741 PMC4263892

[34] Thevissen, K., François, I.E.J.A., Takemoto, J.Y., Ferket, K.K.A., Meert, E.M.K. and Cammue, B.P.A. (2003) DmAMP1, an antifungal plant defensin from dahlia (*Dahlia merckii*), interacts with sphingolipids from *Saccharomyces cerevisiae*. FEMS Microbiol. Lett. 226, 169–173 10.1016/S0378-1097(03)00590-1 13129623

[35] Oeemig, J.S., Lynggaard, C., Knudsen, D.H., Hansen, F.T., Nørgaard, K.D., Schneider, T. et al. (2012) Eurocin, a new fungal defensin: structure, lipid binding, and its mode of action. J. Biol. Chem. 287, 42361–42372 10.1074/jbc.M112.382028 23093408 PMC3516779

[36] Schneider, T., Kruse, T., Wimmer, R., Wiedemann, I., Sass, V., Pag, U. et al. (2010) Plectasin, a fungal defensin, targets the bacterial cell wall precursor lipid II. Science 328, 1168–1172 10.1126/science.1185723 20508130

[37] de Leeuw, E., Li, C., Zeng, P., Li, C., de Buin, M.D., Lu, W.Y. et al. (2010) Functional interaction of human neutrophil peptide-1 with the cell wall precursor lipid II. FEBS Lett. 584, 1543–1548 10.1016/j.febslet.2010.03.004 20214904 PMC3417325

[38] Pridmore, C.J., Rodger, A. and Sanderson, J.M. (2016) The association of defensin HNP-2 with negatively charged membranes: a combined fluorescence and linear dichroism study. Biochim. Biophys. Acta - Biomembr. 1858, 892–903 10.1016/j.bbamem.2016.01.014 26801370

[39] Cools, T.L., Vriens, K., Struyfs, C., Verbandt, S., Ramada, M.H.S., Brand, G.D. et al. (2017) The antifungal plant defensin HsAFP1 is a phosphatidic acid-interacting peptide inducing membrane permeabilization. Front. Microbiol. 8, 2295 10.3389/fmicb.2017.02295 29209301 PMC5702387

[40] Shenkarev, Z.O., Gizatullina, A.K., Finkina, E.I., Alekseeva, E.A., Balandin, S.V., Mineev, K.S. et al. (2014) Heterologous expression and solution structure of defensin from lentil *Lens culinaris*. Biochem. Biophys. Res. Commun. 451, 252–257 10.1016/j.bbrc.2014.07.104 25086358

[41] Ochiai, A., Ogawa, K., Fukuda, M., Suzuki, M., Ito, K., Tanaka, T. et al. (2020) Crystal structure of rice defensin OsAFP1 and molecular insight into lipid-binding. J. Biosci. Bioeng. 130, 6–13 10.1016/j.jbiosc.2020.02.011 32192842

[42] Gonçalves, S., Teixeira, A., Abade, J., De Medeiros, L.N., Kurtenbach, E. and Santos, N.C. (2012) Evaluation of the membrane lipid selectivity of the pea defensin Psd1. Biochim. Biophys. Acta - Biomembr. 1818, 1420–1426 10.1016/j.bbamem.2012.02.012 22373959

[43] Amaral, V.S.G., Fernandes, C.M., Felício, M.R., Valle, A.S., Quintana, P.G., Almeida, C.C. et al. (2019) Psd2 pea defensin shows a preference for mimetic membrane rafts enriched with glucosylceramide and ergosterol. Biochim. Biophys. Acta - Biomembr. 1861, 713–728 10.1016/j.bbamem.2018.12.020 30639288

[44] De Paula, V.S., Razzera, G., Barreto-Bergter, E., Almeida, F.C.L. and Valente, A.P. (2011) Portrayal of complex dynamic properties of sugarcane defensin 5 by NMR: multiple motions associated with membrane interaction. Structure 19, 26–36 10.1016/j.str.2010.11.011 21220113

[45] Parisi, K., Shafee, T.M.A., Quimbar, P., van der Weerden, N.L., Bleackley, M.R. and Anderson, M.A. (2019) The evolution, function and mechanisms of action for plant defensins. Semin. Cell Dev. Biol. 88, 107–118 10.1016/j.semcdb.2018.02.004 29432955

[46] Auten, R.L. and Davis, J.M. (2009) Oxygen toxicity and reactive oxygen species: the devil is in the details. Pediatr. Res. 66, 121–127 10.1203/PDR.0b013e3181a9eafb 19390491

[47] Aerts, A.M., François, I.E.J.A., Meert, E.M.K., Li, Q.T., Cammue, B.P.A. and Thevissen, K. (2007) The antifungal activity of RsAFP2, a plant defensin from *Raphanus sativus*, involves the induction of reactive oxygen species in *Candida albicans*. J. Mol. Microbiol. Biotechnol. 13, 243–247 10.1159/000104753 17827975

[48] Hayes, B.M.E., Bleackley, M.R., Wiltshire, J.L., Anderson, M.A., Traven, A. and Van Der Weerden, N.L. (2013) Identification and mechanism of action of the plant defensin NaD1 as a new member of the antifungal drug arsenal against *Candida albicans*. Antimicrob. Agents Chemother. 57, 3667–3675 10.1128/AAC.00365-13 23689717 PMC3719733

[49] van der Weerden, N.L., Hancock, R.E.W. and Anderson, M.A. (2010) Permeabilization of fungal hyphae by the plant defensin NaD1 occurs through a cell wall-dependent process. J. Biol. Chem. 285, 37513–37520 10.1074/jbc.M110.134882 20861017 PMC2988356

[50] Dudek, J. (2017) Role of cardiolipin in mitochondrial signaling pathways. Front. Cell Dev. Biol. 5, 90 10.3389/fcell.2017.00090 29034233 PMC5626828

[51] Thevissen, K., Ghazi, A., De Samblanx, G.W., Brownlee, C., Osborn, R.W. and Broekaert, W.F. (1996) Fungal membrane responses induced by plant defensins and thionins. J. Biol. Chem. 271, 15018–15025 10.1074/jbc.271.25.15018 8663029

[52] Spelbrink, R.G., Dilmac, N., Allen, A., Smith, T.J., Shah, D.M. and Hockerman, G.H. (2004) Differential antifungal and calcium channel-blocking activity among structurally related plant defensins. Plant Physiol. 135, 2055–2067 10.1104/pp.104.040873 15299136 PMC520777

[53] Muñoz, A., Chu, M., Marris, P.I., Sagaram, U.S., Kaur, J., Shah, D.M. et al. (2014) Specific domains of plant defensins differentially disrupt colony initiation, cell fusion and calcium homeostasis in *Neurospora crassa*. Mol. Microbiol. 92, 1357–1374 10.1111/mmi.12634 24773060

[54] Guzmán-Rodríguez, J.J., López-Gómez, R., Salgado-Garciglia, R., Ochoa-Zarzosa, A. and López-Meza, J.E. (2016) The defensin from avocado (*Persea americana var. drymifolia*) PaDef induces apoptosis in the human breast cancer cell line MCF-7. Biomed. Pharmacother. 82, 620–627 10.1016/j.biopha.2016.05.048 27470405

[55] Dias, C. and Nylandsted, J. (2021) Plasma membrane integrity in health and disease: significance and therapeutic potential. Cell Discov. 7, 1–18 10.1038/s41421-020-00233-2 33462191 PMC7813858

[56] Maik-Rachline, G., Lifshits, L. and Seger, R. (2020) Nuclear P38: roles in physiological and pathological processes and regulation of nuclear translocation. Int. J. Mol. Sci. 21, 1–23 10.3390/IJMS21176102 PMC7504396 32847129

[57] Girisa, S., Shabnam, B., Monisha, J., Fan, L., Halim, C.E., Arfuso, F. et al. (2019) Potential of zerumbone as an anti-cancer agent. Molecules 24, 734 10.3390/molecules24040734 PMC6413012 30781671

[58] Duval, R.E., Grare, M. and Demoré, B. (2019) Fight against antimicrobial resistance: we always need new antibacterials but for right bacteria. Molecules 24, 3152 10.3390/molecules24173152 PMC6749585 31470632

[59] Mühlberg, E., Umstätter, F., Kleist, C., Domhan, C., Mier, W. and Uhl, P. (2020) Renaissance of vancomycin: approaches for breaking antibiotic resistance in multidrug-resistant bacteria. Can. J. Microbiol. 66, 11–16 10.1139/cjm-2019-0309 31545906

[60] Wang, C.H., Hsieh, Y.H., Powers, Z.M. and Kao, C.Y. (2020) Defeating antibiotic-resistant bacteria: exploring alternative therapies for a post-antibiotic era. Int. J. Mol. Sci. 21, 1061 10.3390/ijms21031061 PMC7037027 32033477

[61] Li, J., Koh, J.J., Liu, S., Lakshminarayanan, R., Verma, C.S. and Beuerman, R.W. (2017) Membrane active antimicrobial peptides: translating mechanistic insights to design. Front. Neurosci. 11, 73 10.3389/fnins.2017.00073 28261050 PMC5306396

[62] Abushaheen M.A., M., Fatani, A.J., Alosaimi, M., Mansy, W., George, M. et al. (2020) Antimicrobial resistance, mechanisms and its clinical significance. Dis. Mon. 66, 100971 10.1016/j.disamonth.2020.100971 32201008

[63] McColl, A.I., Bleackley, M.R., Anderson, M.A. and Lowe, R.G.T. (2018) Resistance to the plant defensin NaD1 features modifications to the cell wall and osmo-regulation pathways of yeast. Front. Microbiol. 9, 1648 10.3389/fmicb.2018.01648 30087664 PMC6066574

[64] Baxter, A.A., Poon, I.K.H. and Hulett, M.D. (2017) The lure of the lipids: how defensins exploit membrane phospholipids to induce cytolysis in target cells. Cell Death Dis. 8, e2712 10.1038/cddis.2017.69 28358378 PMC5386563

[65] Phan, T.K., Bindra, G.K., Williams, S.A., Poon, I.K.H. and Hulett, M.D. (2019) Combating human pathogens and cancer by targeting phosphoinositides and their metabolism. Trends Pharmacol. Sci. 40, 866–882 10.1016/j.tips.2019.09.006 31677918

[66] Toledo, J.B., Arnold, M., Kastenmüller, G., Chang, R., Baillie, R.A., Han, X. et al. (2017) Metabolic network failures in Alzheimer's disease: a biochemical road map. Alzheimer's Dement. 13, 965–984 10.1016/j.jalz.2017.01.020 28341160 PMC5866045

[67] van der Veen, J.N., Kennelly, J.P., Wan, S., Vance, J.E., Vance, D.E. and Jacobs, R.L. (2017) The critical role of phosphatidylcholine and phosphatidylethanolamine metabolism in health and disease. Biochim. Biophys. Acta - Biomembr. 1859, 1558–1572 10.1016/j.bbamem.2017.04.006 28411170

[68] Tai, K.P., Kamdar, K., Yamaki, J., Le, V.V., Tran, D., Tran, P. et al. (2015) Microbicidal effects of α- And θ-defensins against antibiotic-resistant *Staphylococcus aureus* and *Pseudomonas aeruginosa*. Innate Immun. 21, 17–29 10.1177/1753425913514784 24345876 PMC4062604

[69] Tai, K.P., Le, V.V., Selsted, M.E. and Ouellette, A.J. (2014) Hydrophobic determinants of α-defensin bactericidal activity. Infect. Immun. 82, 2195–2202 10.1128/IAI.01414-13 24614658 PMC4019156

[70] Lv, C., Han, Y., Yang, D., Zhao, J., Wang, C. and Mu, C. (2020) Antibacterial activities and mechanisms of action of a defensin from manila clam *Ruditapes philippinarum*. Fish Shellfish Immunol. 103, 266–276 10.1016/j.fsi.2020.05.025 32439511

[71] Guzmán-Rodríguez, J.J., López-Gómez, R., Suárez-Rodríguez, L.M., Salgado-Garciglia, R., Rodríguez-Zapata, L.C., Ochoa-Zarzosa, A. et al. (2013) Antibacterial activity of defensin PaDef from avocado fruit (*Persea americana var. drymifolia*) expressed in endothelial cells against *Escherichia coli* and staphylococcus aureus. Biomed. Res. Int. 2013, 986273 10.1155/2013/986273 24319695 PMC3844270

[72] Skalska, J., Andrade, V.M., Cena, G.L., Harvey, P.J., Gaspar, D., Mello érica, O. et al. (2020) Synthesis, structure, and activity of the antifungal plant defensin PvD1. J. Med. Chem. 63, 9391–9402 10.1021/acs.jmedchem.0c00543 32787086

[73] Gonçalves, S., Silva, P.M., Felício, M.R., de Medeiros, L.N., Kurtenbach, E. and Santos, N.C. (2017) Psd1 effects on *Candida albicans* planktonic cells and biofilms. Front. Cell Infect. Microbiol. 7, 249 10.3389/fcimb.2017.00249 28649561 PMC5465278

[74] Vriens, K., Cools, T.L., Harvey, P.J., Craik, D.J., Braem, A., Vleugels, J. et al. (2016) The radish defensins RsAFP1 and RsAFP2 act synergistically with caspofungin against *Candida albicans* biofilms. Peptides 75, 71–79 10.1016/j.peptides.2015.11.001 26592804

[75] Zhu, C., Tan, H., Cheng, T., Shen, H., Shao, J., Guo, Y. et al. (2013) Human β-defensin 3 inhibits antibiotic-resistant *Staphylococcus* biofilm formation. J. Surg. Res. 183, 204–213 10.1203/10.1016/j.jss.2012.11.048 23273885

[76] Riool, M., de Breij, A., Drijfhout, J.W., Nibbering, P.H. and Zaat, S.A.J. (2017) Antimicrobial peptides in biomedical device manufacturing. Front. Chem. 5, 63 10.3389/fchem.2017.00063 28971093 PMC5609632

[77] Mookherjee, N., Anderson, M.A., Haagsman, H.P. and Davidson, D.J. (2020) Antimicrobial host defence peptides: functions and clinical potential. Nat. Rev. Drug Discov. 19, 311–332 10.1038/s41573-019-0058-8 32107480

[78] Bakovic, A., Risner, K., Bhalla, N., Alem, F., Chang, T.L., Weston, W.K. et al. (2021) Brilacidin demonstrates inhibition of SARS-CoV-2 in cell culture. Viruses 13, 271 10.3390/v13020271 33572467 PMC7916214

[79] Andes, D., Craig, W., Nielsen, L.A. and Kristensen, H.H. (2009) In vivo pharmacodynamic characterization of a novel plectasin antibiotic, NZ2114, in a murine infection model. Antimicrob. Agents Chemother. 53, 3003–3009 10.1128/AAC.01584-08 19414576 PMC2704636

[80] Wang, X., Zhang, H. and Chen, X. (2019) Drug resistance and combating drug resistance in cancer. Cancer Drug Resist. 2, 141–160 10.20517/cdr.2019.10 34322663 PMC8315569

[81] Nurgali, K., Jagoe, R.T. and Abalo, R. (2018) Editorial: adverse effects of cancer chemotherapy: anything new to improve tolerance and reduce sequelae? Front. Pharmacol. 9, 245 10.3389/fphar.2018.00245 29623040 PMC5874321

[82] Freites-Martinez, A., Shapiro, J., Goldfarb, S., Nangia, J., Jimenez, J.J., Paus, R. et al. (2019) Hair disorders in patients with cancer. J. Am. Acad. Dermatol. 80, 1179–1196 10.1016/j.jaad.2018.03.055 29660422 PMC6186204

[83] Zhang, Z., Zhou, L., Xie, N., Nice, E.C., Zhang, T., Cui, Y. et al. (2020) Overcoming cancer therapeutic bottleneck by drug repurposing. Signal Transduct. Target Ther. 5, 1–25 10.1038/s41392-020-00213-8 32616710 PMC7331117

[84] Miles, M.A. and Hawkins, C.J. (2020) In vitro analysis reveals necroptotic signaling does not provoke DNA damage or HPRT mutations. Cell Death Dis. 11, 680 10.1038/s41419-020-02879-y 32826875 PMC7442655

[85] Oppelt, A., Haugsten, E.M., Zech, T., Danielsen, H.E., Sveen, A., Lobert, V.H. et al. (2014) PIKfyve, MTMR3 and their product PtdIns5P regulate cancer cell migration and invasion through activation of Rac1. Biochem. J. 461, 383–390 10.1042/BJ20140132 24840251

[86] Thorpe, L.M., Spangle, J.M., Ohlson, C.E., Cheng, H., Roberts, T.M., Cantley, L.C. et al. (2017) PI3K-p110α mediates the oncogenic activity induced by loss of the novel tumor suppressor PI3K-p85α. Proc. Natl Acad. Sci. U.S.A. 114, 7095–7100 10.1073/pnas.1704706114 28630349 PMC5502636

[87] Szlasa, W., Zendran, I., Zalesińska, A., Tarek, M. and Kulbacka, J. (2020) Lipid composition of the cancer cell membrane. J. Bioenerg. Biomembr. 52, 321–342 10.1007/s10863-020-09846-4 32715369 PMC7520422

[88] Alves, A.C., Ribeiro, D., Nunes, C. and Reis, S. (2016) Biophysics in cancer: the relevance of drug-membrane interaction studies. Biochim. Biophys. Acta - Biomembr. 1858, 2231–2244 10.1016/j.bbamem.2016.06.025 27368477

[89] Cui, W., Li, S.Y., Du, J.F., Zhu, Z.M. and An, P. (2012) Silencing phospholipid scramblase 1 expression by RNA interference in colorectal cancer and metastatic liver cancer. Hepatobiliary Pancreat. Dis. Int. 11, 393–400 10.1016/S1499-3872(12)60197-0 22893466

[90] Zhao, Y., Yuan, X., Li, X. and Zhang, Y. (2019) Resveratrol significantly inhibits the occurrence and development of cervical cancer by regulating phospholipid scramblase 1. J. Cell Biochem. 120, 1527–1531 10.1002/jcb.27335 30350320

[91] Ran, S., Downes, A. and Thorpe, P.E. (2002) Increased exposure of anionic phospholipids on the surface of tumor blood vessels. Cancer Res. 62, 6132–6140 PMID: 12414638

[92] Parvy, J.P., Yu, Y., Dostalova, A., Kondo, S., Kurjan, A., Bulet, P. et al. (2019) The antimicrobial peptide defensin cooperates with tumour necrosis factor to drive tumour cell death in *Drosophila*. eLife 8, e45061 10.7554/eLife.45061 31358113 PMC6667213

[93] Flores-Alvarez, L.J., Guzmán-Rodríguez, J.J., López-Gómez, R., Salgado-Garciglia, R., Ochoa-Zarzosa, A. and López-Meza, J.E. (2018) Padef defensin from avocado (*Persea americana var. drymifolia*) is cytotoxic to K562 chronic myeloid leukemia cells through extrinsic apoptosis. Int. J. Biochem. Cell Biol. 99, 10–18 10.1016/j.biocel.2018.03.013 29559362

[94] Figueira, T.N., Oliveira, F.D., Almeida, I., Mello, É.O, Gomes, V.M., Castanho, M.A.R.B. et al. (2017) Challenging metastatic breast cancer with the natural defensin PvD1. Nanoscale 9, 16887–16899 10.1039/c7nr05872a 29076508

[95] Rady, I., Siddiqui, I.A., Rady, M. and Mukhtar, H. (2017) Melittin, a major peptide component of bee venom, and its conjugates in cancer therapy. Cancer Lett. 402, 16–31 10.1016/j.canlet.2017.05.010 28536009 PMC5682937

[96] Pandurangi, R.S., Karwa, A., Sagaram, U.S. and Shah, D. (2021) *Medicago sativa* Defensin 1 (MsDef1), a natural tumor targeted sensitizer for improving chemotherapy: translation from anti-fungal agent to potential anti-cancer agent. bioRxiv, 2021.02.13.431112 10.1101/2021.02.13.431112 PMC10251204 37305575

[97] Lehrer, R.I., Bevins, C.L. and Ganz, T. (2005). Defensins and other antimicrobial peptides and proteins. Mucosal Immunology, 95–110 10.1016/B978-012491543-5/50010-3

[98] Koeninger, L., Armbruster, N.S., Brinch, K.S., Kjaerulf, S., Andersen, B., Langnau, C. et al. (2020) Human β-Defensin 2 mediated immune modulation as treatment for experimental colitis. Front. Immunol. 11, 93 10.3389/fimmu.2020.00093 32076420 PMC7006816

[99] Fruitwala, S., El-Naccache, D.W. and Chang, T.L. (2019) Multifaceted immune functions of human defensins and underlying mechanisms. Semin Cell Dev. Biol. 88, 163–172 10.1016/j.semcdb.2018.02.023 29501617 PMC6485945

[100] Kerenga, B.K., McKenna, J.A., Harvey, P.J., Quimbar, P., Garcia-Ceron, D., Lay, F.T. et al. (2019) Salt-tolerant antifungal and antibacterial activities of the corn defensin ZmD32. Front. Microbiol. 10, 795 10.3389/fmicb.2019.00795 31031739 PMC6474387

[101] Bleackley, M.R., Dawson, C.S., Payne, J.A.E., Harvey, P.J., Rosengren, K.J., Quimbar, P. et al. (2019) The interaction with fungal cell wall polysaccharides determines the salt tolerance of antifungal plant defensins. Cell Surf. 5, 100026 10.1016/j.tcsw.2019.100026 32743142 PMC7389181

[102] Homayun, B., Lin, X. and Choi, H.J. (2019) Challenges and recent progress in oral drug delivery systems for biopharmaceuticals. Pharmaceutics 11, 129 10.3390/pharmaceutics11030129 PMC6471246 30893852

[103] Sharma, A., Vaghasiya, K., Ray, E. and Verma, R.K. (2018) Nano-encapsulated HHC10 host defense peptide (HDP) reduces the growth of *Escherichia coli* via multimodal mechanisms. Artif. Cells Nanomed. Biotechnol. 46, S156–S165 10.1080/21691401.2018.1489823 30032649

